# DL0410 Alleviates Memory Impairment in D-Galactose-Induced Aging Rats by Suppressing Neuroinflammation via the TLR4/MyD88/NF-*κ*B Pathway

**DOI:** 10.1155/2021/6521146

**Published:** 2021-10-04

**Authors:** Baoyue Zhang, Wenwen Lian, Jun Zhao, Zhe Wang, Ailin Liu, Guanhua Du

**Affiliations:** ^1^State Key Laboratory of Bioactive Substances and Functions of Natural Medicines, Institute of Materia Medica, Chinese Academy of Medical Sciences and Peking Union Medical College, Beijing 100050, China; ^2^Beijing Key Laboratory of Drug Target Identification and Drug Screening, Institute of Materia Medica, Chinese Academy of Medical Sciences and Peking Union Medical College, Beijing 100050, China

## Abstract

Oxidative stress and neuroinflammation have been demonstrated to be linked with Alzheimer's disease (AD). In this study, we examined the protective effects of DL0410 in aging rats and explored the underlying mechanism against oxidative damage and neuroinflammation, which was then validated in LPS-stimulated BV2 microglia. We firstly investigated the improvement effects of DL0410 on learning and memory abilities and explored the potential mechanisms in D-gal-induced aging rats. An 8-week treatment with DL0410 significantly improved the learning and cognitive function of D-gal-stimulated Alzheimer's-like rats in the Morris water maze test, step-down test, and novel object recognition test, and the therapeutic effect of DL0410 at 10 mg/kg was even better than that of donepezil. What is more, the results showed that DL0410 alleviated neuron injury, increased the number of synapses, and improved the level of postsynaptic density protein 95 (PSD95) in the hippocampus and cortex. Next, we examined the protective effects of DL0410 against oxidative damage and neuroinflammation. Our observations indicated that DL0410 reduced the production of harmful oxidation products and promoted the antioxidative system, decreased the levels of proinflammatory cytokines, including tumor necrosis factor *α* (TNF-*α*), interleukin 1*β* (IL-1*β*), and interleukin 6 (IL-6), and increased anti-inflammatory cytokines IL-10. Moreover, DL0410 inhibited the activation of astrocytes and microglia and suppressed the activation of the TLR4/MyD88/NF-*κ*B signaling pathway. The anti-inflammation effect of DL0410 was further confirmed in LPS-stimulated BV2 cells, and the results showed that DL0410 reduced the level of inflammatory factors and inhibited the activation of the TLR4/MyD88/TRAF6/NF-*κ*B signaling pathway in BV2 microglia. Molecular docking results indicated that DL0410 occupied the LPS recognition site in the TLR4/MD2 complex. Furthermore, the enhanced expression of claudin-1, claudin-5, occludin, CX43, and ZO-1 indicated that DL0410 protected the blood-brain barrier (BBB) integrity. Together, these results suggest that DL0410 exerts neuroprotective effects against hippocampus and cortex injury induced by D-galactose, and the possible mechanisms include antioxidative stress, antineuroinflammation, improving synaptic plasticity, and maintaining BBB integrity, which is mediated by the TLR4/MyD88/NF-*κ*B signaling pathway inhibition. We suggest that DL0410 is a promising candidate for AD treatment.

## 1. Introduction

Alzheimer's disease (AD) is the most common form of dementia in the elderly and one of the leading contributors to global disease burden. The prevalence of AD is constantly increasing along with the aging population and lack of effective treatment. It is estimated that about 152 million people will suffer from this disease globally by 2050 [1]. A large body of evidence from patients with AD and animal models implies the neuropathological hallmarks of AD including neural loss, synapses loss, amyloid plaques, neurofibrillary tangles (NFTs), oxidative stress, and neuroinflammation [2], which eventually result in neuronal death and cognitive impairment. Although the theory of amyloid plaques and neurofibrillary tangles contributes to understanding and identifying possible therapeutic targets of AD, related therapeutics end in failure, and there is no cure for AD [3, 4]. More and more attention has been attracted by other cellular biological process to find an effective way to deal with the unknown disease.

Oxidative stress is a major factor in aging, cardiovascular disorder, diabetes mellitus, and neurodegenerative disease [5]. Reactive oxygen species- (ROS-) induced oxidative stress and the modification of protein, lipids, and other biomolecules mediated by ROS will lead to impaired cellular function, which was considered an important causative involvement of neuronal death in multiple age-associated neurodegenerative diseases [6]. D-Galactose-stimulated oxidative stress leads to the accumulation of harmful oxidation products [7] and has the potential to cause microglial overactivation, which can induce neuroinflammatory responses by the release of proinflammatory cytokines, including interleukin- (IL-) 1*β*, IL-6, and tumor necrosis factor (TNF-*α*) and the upregulation of cyclooxygenase2 (COX2) and inducible nitric oxide synthase (iNOS) [8]. What is more, ROS generation can be increased by the inflammatory response, and the vicious circle could exacerbate neurodegeneration in the brain [9]. Accumulating evidence demonstrates that systemic chronic exposure to D-gal enhances memory loss and cognitive impairment in rodents [10]. D-gal-induced oxidative damage and neuroinflammation in animal models can well mimic natural aging and have been used in antiaging research worldwide [11, 12].

Basically, the blood-brain barrier (BBB) is composed of capillary endothelial cells, astrocytes, pericytes, neurons, and tight junctions (TJs), which are essential to maintain a homeostatic microenvironment for the structure and function of the brain [13]. BBB breakdown has been observed in older people and in late-onset Alzheimer's disease and is inextricably linked with neuroinflammation [14]. During AD pathology, the activation of microglia in response to oxidative stress is one of the main chronic sources of ROS, which leads to neurotoxicity and BBB function impairment, including the reorganization of TJs [15]. What is more, under some pathological conditions, BBB junction complex is destroyed, which is related to the changes of immune state of central nervous system. Many bioactive molecules are closely related to the destruction of BBB, including proinflammatory cytokines released from overactivated microglia [16]. Astrocytes are able to improve BBB function and maintain tighter TJs (physical barrier), the expression and polarized localization of transporters (transport barrier), and specialized enzymes (metabolic barrier). Astrocytic dysfunction could easily damage the normal physiological function of BBB, inducing the imbalance of A*β* clearance from the brain parenchyma into the blood [17].

**Figure 1:**
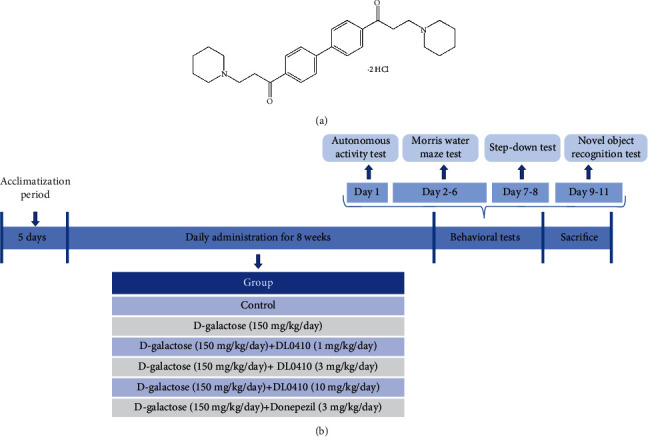
Chemical structure of DL0410 and experiment timeline. (a) Chemical structure of DL0410. (b) Experimental design of drug treatment in SD rats and behavioral analysis.

DL0410 ((1,1′-([1,1′-biphenyl]-4,4′-diyl)bis(3-(piperidin-1-yl)propan-1-one)dihydrochloride) is a multitarget-directed compound that was selected from over 100,000 compounds through a high-throughput screening platform for acetylcholinesterase (AChE) inhibitors, butyrylcholinesterase (BuChE) inhibitors, and histamine H3 receptor (H3R) antagonists [18, 19], and its chemical structure is shown in Figure 1(a). DL0410 treatment significantly improved memory loss and cognitive defects in some AD-related animal models, including scopolamine-induced amnesia mice [20] and APPswe/PSEN1dE9 (APP/PS1) mice [21]. In addition, experimental results indicated that DL0410 had strong antioxidative damage and antineuroinflammation effects in H_2_O_2_-induced SH-SY5Y cells [22]. However, the effects and potential mechanisms of DL0410 against oxidative damage and neuroinflammation in animal models have not been fully explored. In order to deepen the role of DL0410 in AD treatment, D-galactose-induced aging rat model was established in the present study, the improvement effects of DL0410 on learning memory, oxidative damage, neuroinflammation, synaptic plasticity, and BBB damage were investigated in this model, and the possible mechanisms were explored as well. What is more, the anti-inflammation effect was verified again in LPS-stimulated BV2 microglia.

## 2. Material and Methods

### 2.1. Drugs and Reagents

DL0410 (purity ≥ 98% according to high-performance liquid chromatography (HPLC) detection) was provided by the Institute of Materia Medica, Chinese Academy of Medical Sciences (Beijing, China). The detailed information of the obtention of DL0410 was provided in the supplementary materials (available here). Donepezil was provided by Shandong Jinan Dexinjia Biotechnology (Jinan, China). D-Galactose, dimethyl sulfoxide (DMSO), 3-(4,5-dimethyl-2-thiazolyl)-2,5-diphenyl-2-H-tetrazolium bromide (MTT), bovine serum albumin (BSA), lipopolysaccharide (LPS), and 2′,7′-dichlorofluorescin diacetate (H2DCFDA) were the products from Sigma–Aldrich (St. Louis, MO, USA). Lipid peroxidation MDA assay kit, 4% paraformaldehyde (PFA) fix solution, catalase assay kit, and total superoxide dismutase assay kit with WST-8 (#S0103) were the products of Beyotime Institute of Biotechnology (Shanghai, China). TNF-*α* (#ER006, #EH009), IL-1*β* (#ER008, #EH001), IL-6 (#ER003, #EH004), and IL-10 (#ER004) ELISA kits were bought from Excellbio Technology Inc. (Shanghai, China). The rat advanced glycation end products (AGEs) ELISA Kit (#CSB-E09413r) was obtained from CUSABIO Technology (Houston, TX, USA). The BCA protein assay kit was bought from Thermo Fisher Scientific, Inc. (Waltham, MA, USA). The radio immune precipitation assay (RIPA) buffer, primary antibodies against *β*-actin (#3700), inducible nitric oxide synthase (iNOS) (#13120), cyclo-oxygenase-2 (COX2) (#12282), MyD88 (#4283), TRAF6 (#8028), NF-*κ*B p65 (#8242), phosphorylated- (p-) NF-*κ*B p65 (#3033), Histone H3 (#4499), I*κ*B*α* (#4814), p-I*κ*B*α* (#2859), ionized calcium-binding adapter molecule 1 (Iba-1) (#17198), occludin (#91131), claudin-1 (#13255), neuronal nuclear antigen (NeuN) (#24307), SOD1 (#37385), and SOD2 (#13141) were the products of Cell Signaling Technology (Danvers, MA, USA). Primary antibody against claudin-5 (#AF5216) was provided by Affinity Biosciences (Cincinnati, OH, USA). Primary antibody against Toll-like receptor 4 (TLR4) (19811-1-AP) was purchased from the ProteinTech Group (Manchester, United Kingdom). Primary antibodies against postsynaptic density protein 95 (PSD95) (#ab18258) and glial fibrillary acidic protein (GFAP) (#ab7260) were purchased from Abcam (Cambridge, United Kingdom). Primary antibody against connexin 43 (CX43) (#ER1802-88) was the product of Hangzhou HuaAn Biotechnology Co., Ltd. (Hangzhou, China). Primary antibody against zona occludens protein 1 (ZO-1) (#YN1410) was purchased from the Immunoway Biotechnology Company. The protease inhibitor cocktail and phosphatase inhibitor cocktail were obtained from Kangwei Biotechnology (Beijing, China). Fetal bovine serum (FBS), Dulbecco's modified Eagle's medium (DMEM) and phosphate-buffered saline (PBS) were provided by Gibco (Carlsbad, California, USA). The nitric oxide (NO) assay kit was provided by Applygen Technologies Inc. (Beijing, China).

### 2.2. Animals and Treatment

The Sprague-Dawley (SD) male rats (7 weeks old, 210–270 g average body weight) were purchased from Vital River Laboratory Animal Technology (Beijing, China). The animals were housed in an air-conditioned room (temperature 23°C ± 2°C and humidity of 50% ± 10%), under a 12-h light/dark cycle, with free access to food and water. All the experimental procedures were undertaken in accordance with the Guide for the Care and Use of Laboratory Animals (National Institutes of Health, Bethesda, MD, USA) and approved by the Animal Care and Use Ethics Committee of Peking Union Medical College and Chinese Academy of Medical Sciences (Beijing, China) (No. 00005665, Date 15th Mar 2019). The experimental animals were divided randomly into the following six groups (*n* = 12 rats/group): control group, D-galactose model group (D-gal group), D-galactose+DL0410-1 mg/kg group (D-gal+DL1 group), D-galactose+DL0410-3 mg/kg group (D-gal+DL3 group), D-galactose+DL0410-10 mg/kg group (D-gal+DL10 group), and D-galactose+donepezil-3 mg/kg group (D-gal+Don3 group).

#### 2.2.1. Drug Preparation and Treatment

D-Galactose was dissolved in sterile saline. DL0410 and donepezil were dissolved in distilled water.

1. Control (CTL) group: rats treated with saline (s.c.) in the neck + distilled water (1 mg/kg/day, p.o.) for 8 weeks
2. D-gal group: rats treated with D-gal (150 mg/kg/day, s.c.) in the neck + distilled water (1 mg/kg/day, p.o.) for 8 weeks
3. D-gal+DL1 group: rats treated with D-gal (150 mg/kg/day, s.c.) + DL0410 (1 mg/kg/day, p.o.) for 8 weeks
4. D-gal+DL3 group: rats treated with D-gal (150 mg/kg/day, s.c.) + DL0410 (3 mg/kg/day, p.o.) for 8 weeks
5. D-gal+DL10 group: rats treated with D-gal (150 mg/kg/day, s.c.) + DL0410 (10 mg/kg/day, p.o.) for 8 weeks
6. D-gal+Don3 group: rats treated with D-gal (150 mg/kg/day, s.c.) + donepezil (3 mg/kg/day, p.o.) for 8 weeks.

### 2.3. Behavioral Tests

After treatment with D-gal and DL0410 for eight weeks, the behavioral study of the experimental animals (*n* = 12/group) was carried out by the autonomous activity test, the Morris water maze test, and followed by the step-down test and the novel object recognition test. The detailed experiment timeline is shown in Figure 1(b).

#### 2.3.1. Autonomous Activity Test

The autonomous activity test was performed after eight weeks' daily administration of D-galactose and DL0410/donepezil. The experiment was carried out in a ZIL-2 rat autonomous activity device, including six infrared detectors around each black box (40 cm in diameter and 21 cm in height). When the rat was moving in the box, it can be sensed by the infrared detector and recorded as 1 time. A rat was put into the box to adapt itself to the environment for 1 min, and then autonomous activity within 5 min was recorded.

#### 2.3.2. The Morris Water Maze Test

The Morris water maze test was performed to detect the learning and memory ability of rats in each group. The water maze apparatus is composed of a circular water tank (150 cm in diameter and 50 cm in height) and automatic recording system. The tank was filled with water to a depth of 31 cm and divided equally into four quadrants, and an acrylic platform (13 cm in diameter and 30 cm in height) hidden 1 cm below the water surface was placed in the first quadrant. After administration, the Morris water maze test was conducted for 4 consecutive days, and each rat was trained for 60 s each time, twice a day. During the training period, if the rats found and climbed to the platform within 60 s, they were allowed to stay on the platform for 15 s; if the rats failed to find or climb to the platform within 1 min, they were guided to the platform and stayed for 15 s. The latency of the first arrival of the rats in each group within 1 min was recorded. The space exploration experiment was carried out on the fifth day. The acrylic platform was removed, and the latency, number of crossing the platform, and the residence time in the target quadrant of the rats within 2 minutes were recorded and analyzed.

#### 2.3.3. Step-Down Test

The learning and memory functions of experimental animals were further evaluated by the step-down test. The experimental device is composed of an acrylic box (30 cm × 30 cm × 30 cm) with a stainless-steel grid floor and a rubber platform (8 cm in diameter and 4 cm in height) placed in the center of the floor. In the experiment, the rats were first placed on the stainless-steel grid floor, and after being electrified, the rats were stimulated with electric shock. The normal reaction was to jump on the platform to avoid electric shock. Most rats might jump off the platform again or several times, and they would quickly jump back to the platform when once hurt by electric shock. After 24 hours, the rats were put on the platform, and the latency to step down (four paws on the grid) for the first time and the number of error times reacting to shocks within 5 min were recorded for the reference of learning performances.

#### 2.3.4. Novel Object Recognition (NOR) Test

The novel object recognition test was performed in an open field (50 cm × 50 cm wide × 40 cm high). Rats were given a 5 min habituation trial with no objects in the training box on the first day (habituation session). The sampling session is 24 hours after habituation session, during which period the rats were gently placed into the open field and allowed to freely explore two identical objects placed in two opposite areas of the behavior box for 5 min. On the third day of test session, one object remained unchanged (familiar object), and the other object was replaced by a new object (novel object), and the rats were put into the behavior box again to explore for 5 minutes without limitation, and the time rats spent on exploring the familiar object and novel object during test session was recorded as *F* and *N*, respectively. The discrimination index was defined as *N*/(*F* + *N*) × 100%.

### 2.4. Collection of Brain Tissue and Protein Extraction

After drug administration and behavior tests, the rats were sacrificed by decapitation under general anesthesia, and the brains were removed immediately. The cortex and hippocampus were carefully dissected from brains, and all the tissues were frozen in liquid nitrogen and stored at -80°C until use. The mixture of PBS with phosphatase and protease inhibitors was used to homogenize the hippocampus and cortex, followed by 12000 rpm centrifugation at 4°C for 15 minutes (Allegra™ X-22R centrifuge, Beckman Coulter, Brea, CA, USA). The supernatants were collected and stored at -80°C.

### 2.5. MDA Assay

Malondialdehyde (MDA) in tissue homogenate of the hippocampus and cortex was measured by using the MDA assay kit according to the manufacturer's procedure.

### 2.6. SOD Activity Assay

The superoxide dismutase (SOD) activity was detected based on its capacity to competitively inhibit the combination of WST-8 and superoxide radicals generated by xanthine oxidase, using a Cu/Zn-SOD and Mn-SOD assay kit with WST-8 according to the manufacturer's instructions. In short, the samples of different dilutions, detection buffer, WST-8 solution, and the starting solution were added to the transparent 96-well plate successively. The plate was incubated at 37°C for 30 minutes, and the absorbance at 450 nm was read (SpectraMax M5, Molecular Devices, USA). The activity of SOD was averaged by protein concentration.

### 2.7. Catalase Activity Assay

The samples, buffer, and hydrogen peroxide (H_2_O_2_) were added to the microcentrifuge tube, and the mixed solution was incubated at 25°C for 5 minutes. The reaction was terminated after the addition of stop solution. 40 *μ*L detection buffer was added in a clean centrifuge tube, and mixed with 10 *μ*L of the terminated reaction system. Subsequently, 10 *μ*L mixture was transferred to 96-well microporous plate, and colored solution was added. After incubation at 25°C for 15 minutes, the absorbance at 520 nm was read (SpectraMax M5, Molecular Devices, USA). The catalase activity was averaged by protein concentration.

### 2.8. ELISA Assay

The tissue homogenate of hippocampus and cortex and medium supernatant of BV2 cells were thawed on ice and analyzed by ELISA assay for quantification of AGEs, TNF-*α*, IL-1*β*, IL-6, and IL-10 according to the introductions of the manufacturer (SpectraMax M5, Molecular Devices, USA).

### 2.9. Immunofluorescence Staining

To detect the expression level of NeuN in the brain of D-gal-treated rats, the brain tissue was fixed with 4% paraformaldehyde for immunofluorescence. Shorty, the brain sections were permeabilized with 0.3% Triton X-100 and blocked by serum before incubation with anti-NeuN primary antibody (1 : 400) overnight at 4°C, incubated with appropriate fluorescent probe-conjugated secondary antibody at 25°C for 2 h, and then stained with 4,6-diamidino-2-phenylindole (DAPI) for 10 min to make nuclei stained. The slides were scanned by Pannoramic DESK, P-MIDI, P250 (3DHISTECH Ltd., Hungary).

For immunofluorescence staining of NF-*κ*B p65, BV2 cells were fixed with 4% paraformaldehyde for 30 min at 37°C and then permeabilized by 0.3% Triton X-100 for 15 min at room temperature. After being blocked by 5% BSA for 2 h at room temperature, the fixed cells were incubated with primary antibody against NF-*κ*B p65 overnight at 4°C, followed by incubation with secondary antibody for 2 h at 37°C. At last, the fixed cells were stained with DAPI at room temperature for 15 min, and the images were observed using a fluorescence microscope (ECLIPSE Ti-U, Nikon, Tokyo, Japan).

### 2.10. Immunohistochemistry (IHC) Analysis

The rats were killed and infused with 0.1 M PBS and then stained with 4% PFA fix solution for IHC analysis. Paraffin slides were incubated with anti-PSD95, anti-Iba-1, and anti-GFAP primary antibody (1 : 1000) at 4°C overnight. After being washed with PBS, the slides were incubated with secondary antibody for 2 h at room temperature. Hematoxylin was used for nuclear staining. The slides were scanned by Pannoramic DESK, P-MIDI, P250 (3DHISTECH Ltd., Hungary).

### 2.11. Transmission Electron Microscope (TEM)

To observe the number and morphology of synapses in the brain, the brain issue was immediately fixed with electron microscope solution. 1 mm^3^ of hippocampus was post fixed in 1% osmium tetroxide at 25°C for 2 hours. Samples are gradient dehydrated before being embedded in epoxy resin (TAAB medium grade) and polymerized at 60°C. Sections were cut into ultrathin sections (70 nm) and then were stained with 3% uranyl acetate and lead citrate for 15 minutes. The transmission electron microscope (HITACHI HT 7800, Hitachi High-Tech Co., Tokyo, Japan) was used for image acquisition. Regions of interest were scanned at 5000x, and the number of synapses were counted.

### 2.12. Western Blot Analysis

The primary hippocampus, cortex tissues of rats, and BV2 cells were washed, collected, and lysed in iced RIPA buffer mixed with the cocktail protease inhibitor and phosphatase inhibitor. The supernatant was collected after centrifugation for BCA assay. The same amount of protein for each sample was separated by 8-12% SDS-PAGE and then transferred to polyvinylidene difluoride (PVDF) membranes. The membranes were cut into pieces in accordance with target protein quality and blocked with 5% bovine serum albumin (BSA) solution at room temperature for 2 hours to occupy nonspecific sites. The membranes were incubated with different primary antibodies at 4°C overnight. After being washed with Tris-buffered saline containing 0.1% Tween 20 (TBST) solution, the membranes were exposed to horseradish peroxidase-conjugated secondary antibody incubation for 2 h at room temperature, and the protein bands were visualized by the enhanced electrochemiluminescence (ECL) system (Tanon 5200, Tanon, Shanghai, China), and the Image-Pro Plus software 6.0 (Media Cybernetics, Silver Spring, USA) was employed to analyze the data.

### 2.13. Cell Culture

Immortalized mouse BV2 microglial cell line was provided by Institute of Basic Medical Sciences, Chinese Academy of Medical Sciences & Peking Union Medical College (Beijing, China), and cultured in Dulbecco's modified Eagle's medium (DMEM) supplemented with 10% FBS in a humidified incubator containing 5% CO_2_ at 37°C.

### 2.14. Cell Viability Assay

BV2 cells were seeded into transparent 96-well microplates at a density of 1.2 × 10^4^ cells/well. After 24 h of incubation, cells were pretreated with DL0410 for 2 h at doses of 1-30 *μ*M and then coincubated with or without LPS at 37°C for a further 24 h. 100 *μ*L of MTT (0.5 mg/mL) was added to each well, and the cells were further incubated at 37°C in darkness for 4 h. Then, the supernatant was removed, and 100 *μ*L of DMSO were added to dissolve crystalline formazan. The absorbance at 570 nm was measured using a microplate reader (SpectraMax M5, Molecular Devices, USA).

### 2.15. Nitric Oxide Assay

BV2 cells were seeded in a 96-well cell culture microplate at a density of 1.2 × 104 cells/well and then treated with DL0410 at the given concentration in the presence of LPS (100 ng/mL). 50 *μ*L of medium supernatant was transferred into a new microplate and coincubated with the Griess reagent A (50 *μ*L) and Griess reagent B (50 *μ*L) at 25°C for 5 min in the dark, and then the absorbance was determined at 540 nm with a spectrophotometer plate reader.

### 2.16. Measurement of Intracellular Reactive Oxygen Species (ROS)

BV2 microglia were seeded in 6-well plates and cultured as described previously. After incubation with DL0410 accompanied by stimulation with LPS, the level of intracellular ROS was measured by H2DCFDA fluorescence. Cells in each group were incubated with 2 *μ*M H2DCFDA dissolved in DMED at 37°C for 30 min in the dark, and then washed with PBS for three times. Cells were observed using fluorescence microscope (ECLIPSE Ti-U, Nikon, Tokyo, Japan).

### 2.17. Molecular Docking

The chemical structure of DL0410 was built by ChemDraw, and the crystal structure of human TLR4 cocrystallized with MD2 (PDB ID: 3FXI) was obtained from the Protein Data Bank database (http://www.rcsb.org/). DL0410 was docked into the binding site of TLR4/MD2 with CDOCKER methodology in Discovery Studio version 2018 (San Diego, CA, USA). CDOCKER is an important docking program with the purpose of searching the macromolecular protein for the most favorable binding configurations between a flexible ligand and a rigid receptor in the CHARMm force field. The structure of DL0410 was prepared by adding hydrogen, converting into 3D structure, pH-based ionization, and charge neutralization with the Prepare Ligand module. The 3D structure of TLR4/MD2 was processed by removing water and adding hydrogen, and then the Prepare Protein module was used to correct protein residue connectivity, missing loop regions, and backbone atoms. The prepared structure of TLR4/MD2 was defined as the receptor, and the LPS-binding site was defined as active pocket. The interaction energy for the final TLR4/MD2-ligand complex poses was calculated, and the top coring pose was retained. The binding mode of DL0410 to TLR4/MD2 was visualized by the View Interaction module.

### 2.18. Statistical Analysis

All of the experiment results are expressed as the mean ± SEM. Statistical differences among different groups were assessed with one-way analysis of variance (ANOVA) test using GraphPad Prism 7.00 (GraphPad Software Inc., CA, USA). *p* values less than 0.05 were considered statistically significant.

## 3. Results

### 3.1. DL0410 Reversed Memory Impairment in D-Galactose-Treated Rats

**Figure 2:**
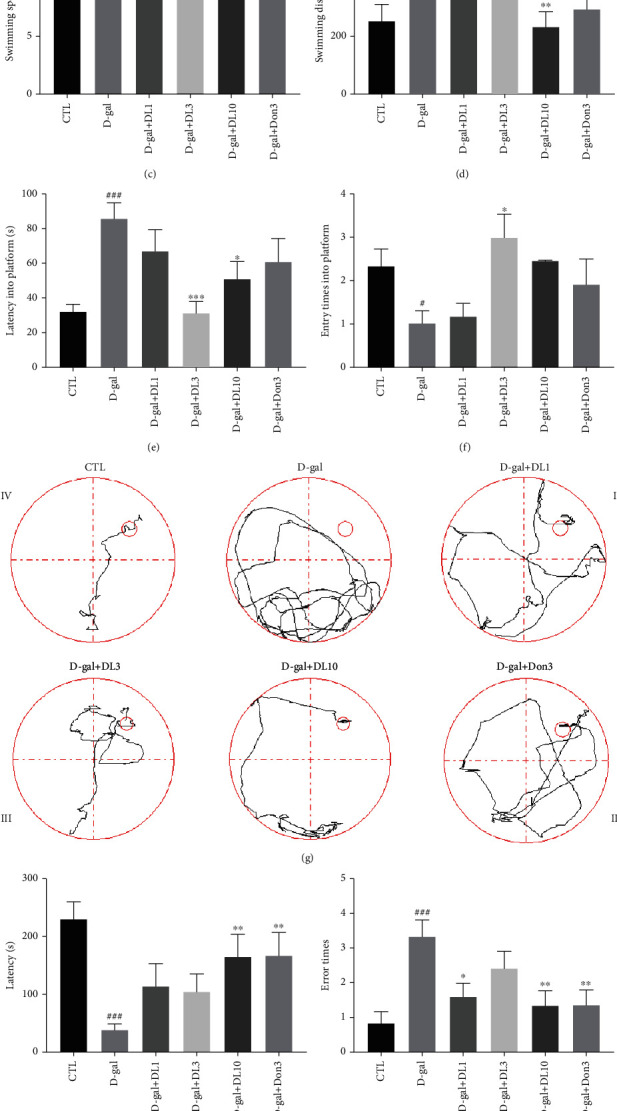
DL0410-improved D-gal weakened learning and memory function in rats. Rats were treated following the methods as described above (*n* = 12). (a) Number of autonomic activities among groups administered D-galactose, donepezil, and DL0410, and there were no notable differences. (b–g) The effect of DL0410 on spatial learning and memory of rats in the MWM test. (b)The rats were trained for four consecutive days, and the escape latency time (s) during training was recorded everyday. (c) Administration of D-galactose, donepezil, and DL0410 had no effect on swimming speed of rats. For the space exploration test, the searching distance (d), latency into platform (e), and the platform area crossings (f) were calculated on day 5. (g) The representative search routes for different groups of rats on the fourth day of the training period. (h, i) The effects of DL0410 on learning and memory of rats in the step-down test. (h) DL0410 was able to prolong the latency of rats stepping onto the stainless-steel grid floor. (i) DL0410 reduced the error times of rats jumping off the rubber platform. (j) The discrimination index during the test session of NOR memory test. Data are the mean ± SEM (*n* = 12). ^#^*p* < 0.05, ^##^*p* < 0.01, ^###^*p* < 0.001 vs. the control group, ^∗^*p* < 0.05, ^∗∗^*p* < 0.01, ^∗∗∗^*p* < 0.001 vs. the model group.

The effects of DL0410 on animal behavior and memory of D-gal-induced Alzheimer's-like rats were studied using the Morris water maze (MWM) test. MWM is a classic test aimed at evaluating the spatial learning and memory ability of rodents [23]. As shown in Figure 2(a), D-galactose, donepezil, and DL0410 had no significant influence on the autonomic activities of rats in each group. During the training period of four days, the mean latency to find the platform was gradually shortened in all groups. However, compared with the rats treated with saline, the rats in the D-gal group showed a longer latency, indicating that the ability of spatial learning and memory was impaired in the rats stimulated with D-gal. Compared with the model group, DL0410 significantly reduced the latency to find the platform during the training days. Subsequently, we analyzed the average swimming speed on the fifth day to compare the movement ability among different groups. The results showed that the swimming speed was not influenced by D-galactose, donepezil, and DL0410, as shown in Figure 2(c). Figures 2(d) and 2(e) show that compared with the normal control group in the space exploration test on day 5, the searching distance and latency of rats treated with D-gal were significantly increased (*p* < 0.01 and *p* < 0.001, respectively), which were notably reduced by DL0410, and the effect of DL0410 was better than that of donepezil in some aspects. Compared with the rats treating with D-gal alone, the times of crossing platform in the middle- and high-dose groups of DL0410 was significantly increased (Figure 2(f)).  Figure 2(g) shows that rats in DL0410-administered groups were able to find the platform more easily and quickly in comparison with those in model group. The step-down test is a passive avoidance task, and step-down latency and error times are the two most important parameters of learning and memory performance [24]. As we can see from Figures 2(h) and 2(i), D-galactose treatment reduced the latency, and the error times of the model group were increased significantly compared with control group. DL0410 and donepezil were able to prolong the latency and cut down on the error times. What is more, as shown in Figure 2(j), DL0410 improved NOR memory as suggested by the obvious increase in the discrimination index of rats. The results of the MWM test, step-down test, and NOR memory test indicated that DL0410 improved spatial cognition, learning, and memory ability of D-gal-induced aging rats.

### 3.2. DL0410 Inhibited Oxidative Damage in D-Galactose-Treated Rats

**Figure 3:**
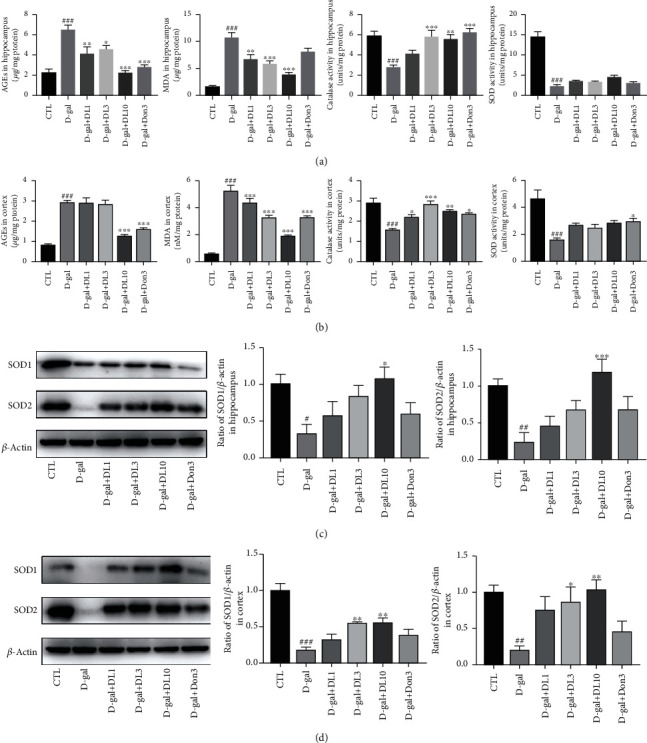
Effects of DL0410 on oxidative damage and antioxidative enzyme activity in the hippocampus and cortex. (a) DL0410 decreased the production and accumulation AGEs and MDA and increased the activity of catalase and superoxide dismutase (SOD) in the hippocampus. (b) DL0410 decreased the levels of AGEs and MDA and strengthened the activity of catalase and SOD in the cortex. (c) The Western blot detection and quantitative analysis of SOD1 and SOD2 expression in the hippocampus. (d) The Western blot detection and quantitative analysis of SOD1 and SOD2 expression in cortex. Data are the mean ± SEM (*n* = 6 − 7). ^###^*p* < 0.001 vs. the control group, ^∗^*p* < 0.05, ^∗∗^*p* < 0.01, ∗∗∗*p* < 0.001 vs. the model group.

D-Galactose is a kind of reducing sugar and could be metabolized to galactitol under the action of aldose reductase, and the latter's metabolic process will be accompanied by the production of a large number of superoxide anions, which will directly damage tissues and organs [25]. To verify whether DL0410 can improve the antioxidant capacity of D-galactose-induced rats, we performed detection on common harmful oxidation products in hippocampal and cortical extractions of each group. As shown in Figure 3, D-galactose treatment significantly stimulated the accumulation of MDA and AGEs, while DL0410 could obviously inhibit this phenomenon. A normal body needs to maintain balance between oxidation system and antioxidant system, of which superoxide dismutases (SODs) and catalase are important candidates. The main function of SODs is to convert superoxide radicals into molecular oxygen and hydrogen peroxide, thus preventing the production of ROS, so it plays a key role in antioxidant reaction. SOD1 and SOD2 are SOD isoenzymes present inside cells, which have been reported to be associated with Alzheimer's disease. We can infer from Figure 3 that D-galactose destroyed the antioxidant system in the rat brain and DL0410 could effectively reduce the damage caused by D-galactose. These results indicated that DL0410 could protect rats against oxidative damage via improving antioxidative defense and preventing the formation of peroxides in the hippocampus and cortex.

### 3.3. DL0410 Improved Neuron Morphology in the Hippocampus and Cortex of D-gal-Stimulated Rats

**Figure 4:**
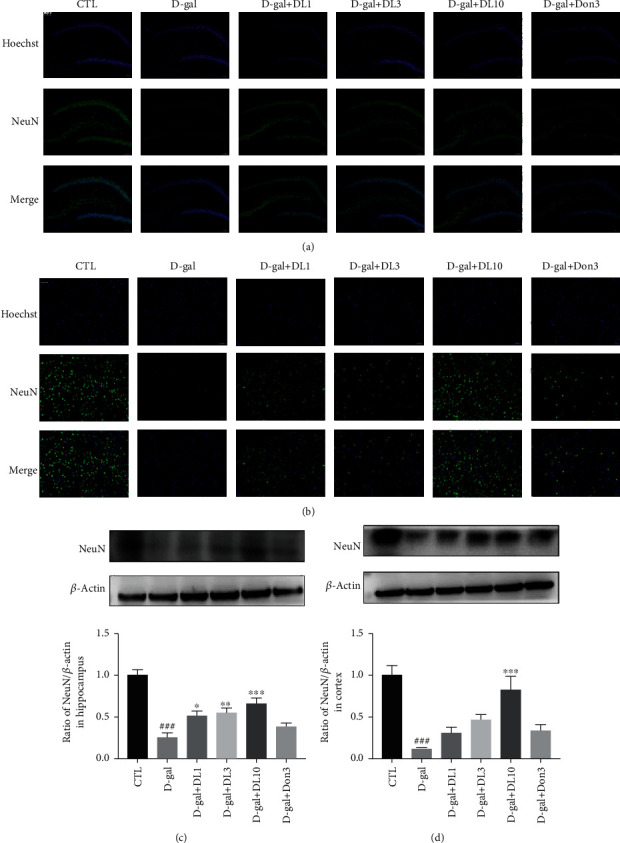
DL0410 treatment-improved D-gal-induced rats decreased expression of NeuN in hippocampus and cortex. (a) Representative images of immunofluorescence staining in hippocampus of rats. (b) Representative images of immunofluorescence staining in cortex of rats. (c) The Western blot detection and quantitative analysis of NeuN expression in hippocampus. (d) The Western blot detection and quantitative analysis of NeuN expression in cortex. Bar = 100 *μ*m. Values are expressed mean ± SEM of six independent experiments. ^###^*p* < 0.001 vs. the control group, ^∗^*p* < 0.05, ^∗∗^*p* < 0.01, ^∗∗∗^*p* < 0.001 vs. the model group.

NeuN, a neuronal specific nuclear protein which locates in the neuron nucleus, is a proverbially used marker to quantify normal neurons and identify the loss of neurons in neurodegenerative disease studies, while degenerating neurons tend to lose this marker [26]. A notable decrease in NeuN staining fluorescence was detected in D-gal-stimulated rats compared with the normal control group, which may indicate that D-galactose stimulated or damaged neurons. However, compared with D-galactose-induced rats, NeuN staining of the hippocampus and cortex was increased after treatment with DL0410 (Figures 4(a) and 4(b)), which was further validated by immunoblot analysis (Figures 4(c) and 4(d)), indicating that DL0410 improved neuron morphology in the hippocampus and cortex of D-gal-stimulated rats.

### 3.4. DL0410 Promoted the Number of Synapses and Structural Synaptic Plasticity of D-gal-Stimulated Rats

**Figure 5:**
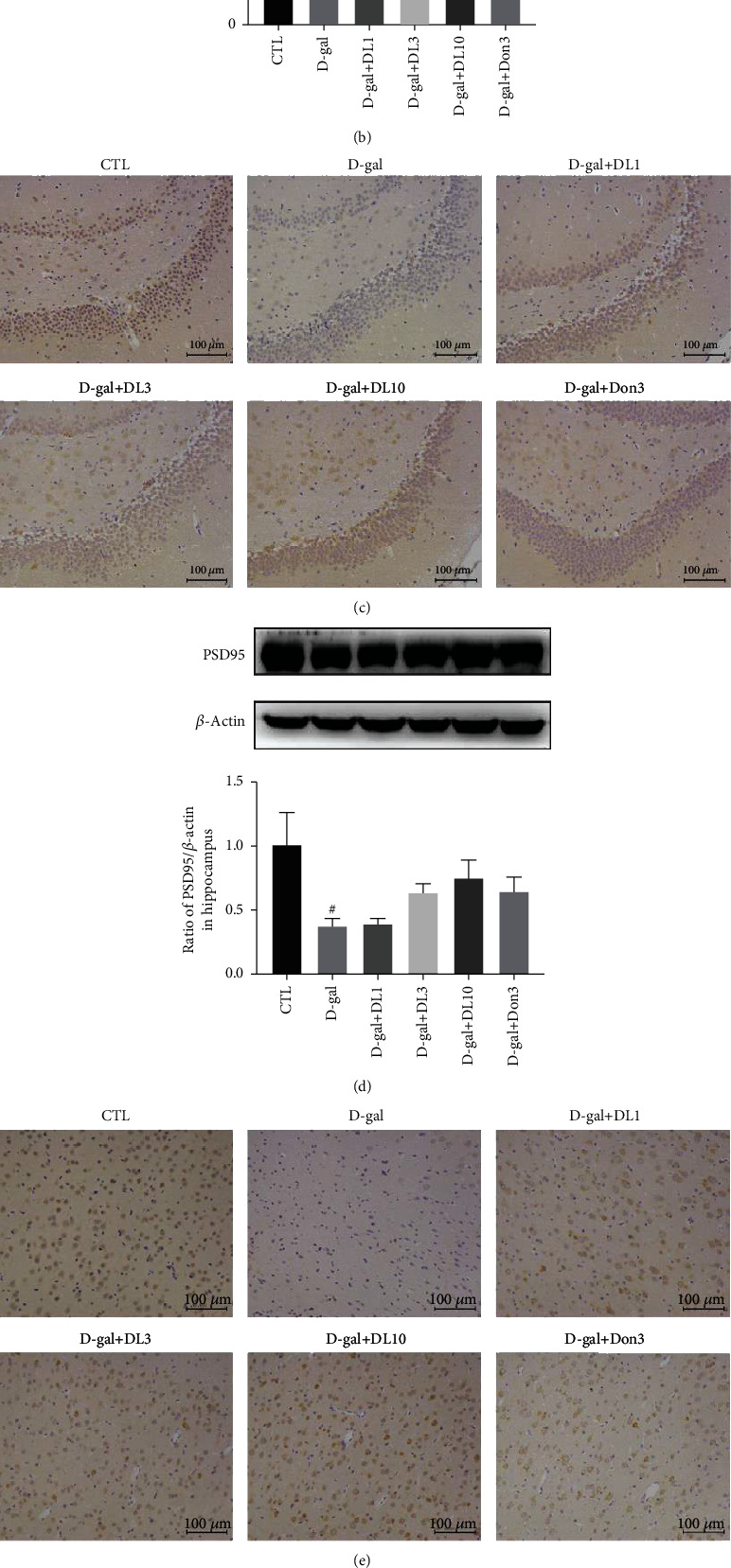
Effects of DL0410 on the number of synapses by transmission electron microscope and the expression of PSD95 stained by IHC and analyzed by Western blot with PSD95. (a) Representative electron micrographs of the hippocampus. The representative synapse structures were pointed out in the images. (b) The effect of DL0410 on the number of synapses in the hippocampus. (c) Representative images of PSD95 IHC in the hippocampus of rats. (d) The Western blot detection of PSD95 expression in the hippocampus, and quantitative analysis of PSD95 in hippocampus. (e) Representative images of PSD95 IHC in the cortex of rats. (f) The Western blot detection of PSD95 expression in the cortex the quantitative analysis of PSD95 in cortex. Bar = 10 *μ*m in transmission electron microscope images, and Bar = 100 *μ*m in IHC images. Values are mean ± SEM (*n* = 6). ^#^p < 0.05, ^##^*p* < 0.01 vs. the control group, ^∗^*p* < 0.05, ^∗∗^*p* < 0.01 vs. the model group.

Postsynaptic density protein 95 (PSD95) is a main scaffold protein in the synapses, which is necessary to maintain synaptic plasticity [27]. Decreased synaptic plasticity and synapse loss are the direct causes of cognitive function impairment [28]. D-gal was shown to be able to decrease the number of synapses, cause damage to synaptic plasticity, and affect the expression of PSD95 [29]. To determine whether DL0410 could improve the phenomenon of synapse loss and increase the expression of PSD95 *in vivo* after D-gal induction, electron microscopy and IHC analysis coupled with Western blotting for PSD95 were performed after 8 weeks' treatment. Compared with D-gal-treated SD rats, DL0410-treated rats had increased number of synapses in the hippocampus (Figures 5(a) and 5(b)). The results of IHC are shown in Figures 5(c) and 5(e). As indicated by the deep brown dyed area of the brain slide, D-gal treatment reduced the expression of PSD95 in the hippocampus and cortex. However, administration with DL0410 increased the number of deep brown dyed cells, suggesting the improved expression of PSD95. The quantification analysis of Western blot also showed that DL0410 could effectively increase the expression of PSD95 that was downregulated by D-gal, indicating that DL0410 promoted the structural synaptic plasticity in the hippocampus and cortex of D-gal-stimulated rats, as shown in Figures 5(d) and 5(f).

### 3.5. DL0410 Inhibited Microglia and Astrocytes Activation in the Hippocampus and Cortex of Rats Induced by D-gal

**Figure 6:**
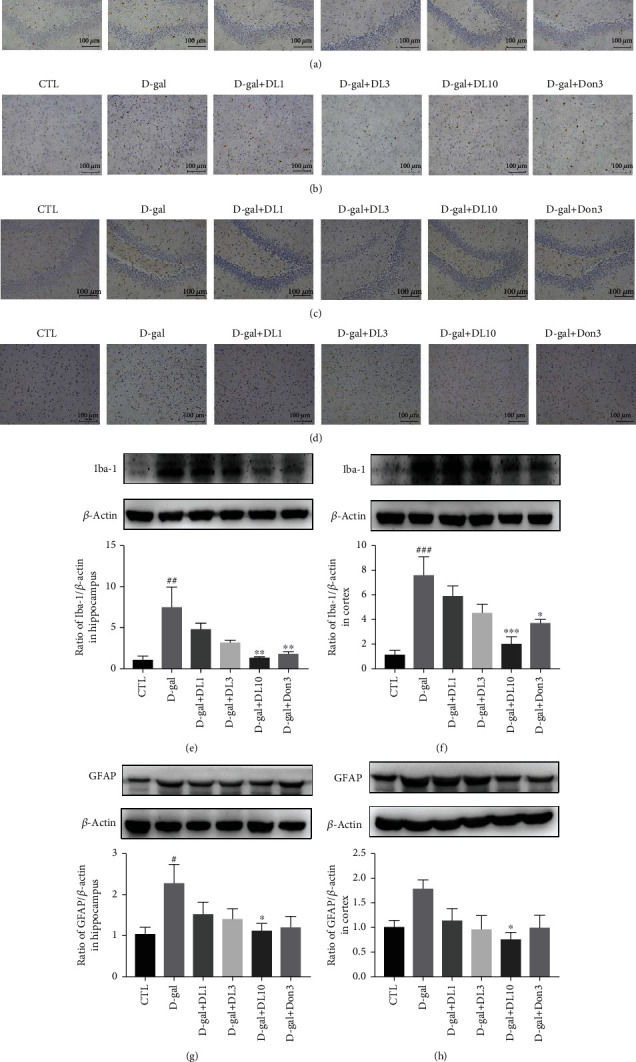
Effect of DL0410 on microglial activation and astrocyte activation stained by IHC and analyzed by Western blot with Iba-1 and GFAP. Representative staining images of Iba-1 IHC staining in the hippocampus (a) and cortex (b) of rats. Representative staining images of GFAP IHC staining in the hippocampus (c) and cortex (d) of rats. The Western blot detection of Iba-1 expression and the quantitative analysis in the hippocampus (e) and cortex (f) of rats. The Western blot detection of GFAP expression and the quantitative analysis in the hippocampus (g) and cortex (h) of rats. Bar = 100 *μ*m. Values are mean ± SEM (*n* = 6). ^#^*p* < 0.05, ^##^*p* < 0.01, ^###^*p* < 0.001 vs. the control group, ^∗^*p* < 0.05, ^∗∗^*p* < 0.01, ^∗∗∗^*p* < 0.001 vs. the model group.

Microglial cells and astrocytes have been characterized as the main cell types in the inflammatory response of central nervous system (CNS). In addition to the amyloid plaques and NFTs, microglial overactivation is a driving force for neurodegeneration. Astrocytes are macroglial cells participating in some major functions including synaptic transmission, synaptogenesis, neurogenesis, and formation and maintenance of the BBB integrity [30]. What is more, astrocytes play an important role in metabolic regulation and ion balancing. Pathologic astrocytes have been identified in some neurological diseases, taking AD, Parkinson's disease (PD), multiple sclerosis (MS), amyotrophic lateral sclerosis (ALS), and epilepsy for example [31]. Iba-1 is a specified marker of microglia. When the nervous system is damaged, microglia will change from resting state to active state, and the higher expression of Iba-1 would be detected. GFAP is a specific marker of astrocytes [32]. The phenomenon of astrocytes activation is usually accompanied by an increased expression of GFAP. In order to observe whether DL0410 could inhibit the activation of microglia and astrocytes, Iba-1 and GFAP IHC staining were performed on the brain sections of rats in each group. Stimulation with D-galactose increased the number of deep brown dyed microglia and astrocytes with long membrane protrusions in the hippocampus and cortex, indicating the overactivation of microglia and astrocytes (Figures 6(a)–6(d)). DL0410 treatment was capable of inhibiting the expression of Iba-1 and GFAP in the hippocampus and cortex of D-galactose-injured rats, which was also supported by the results of Western blot (Figures 6(e)–6(h)). Taken together, all the results showed that DL0410 inhibited the activation of microglia and astrocytes after D-galactose stimulation.

### 3.6. DL0410 Decreased Inflammatory Cytokines and Mediators in the Hippocampus and Cortex of D-gal-Stimulated Rats

**Figure 7:**
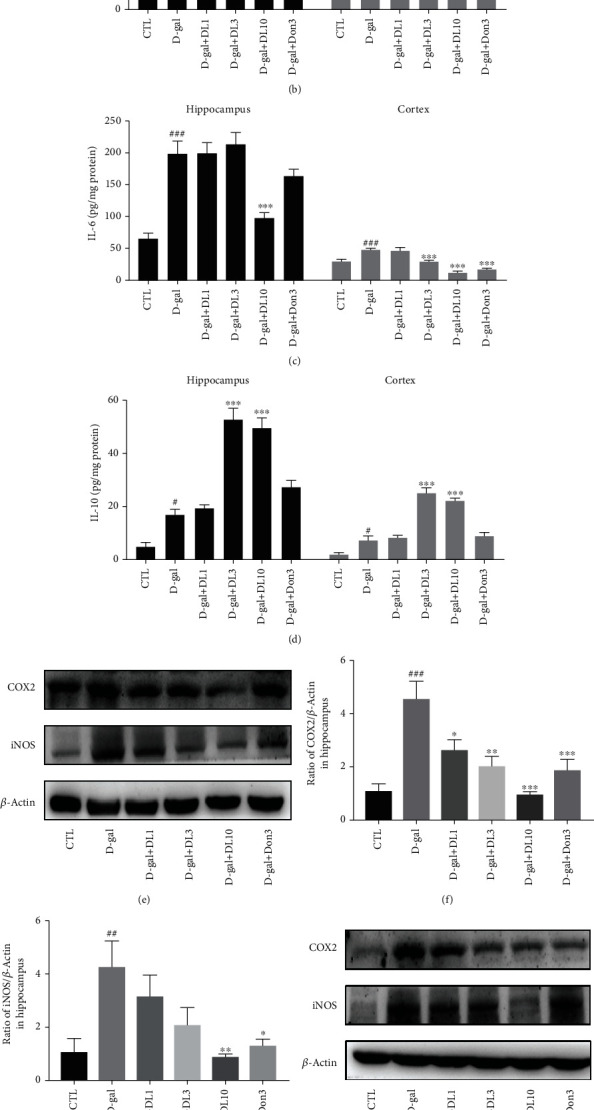
DL0410 decreased the inflammatory factors in the hippocampus and cortex of D-gal-stimulated rats. TNF-*α* (a), IL-1*β* (b), IL-6 (c), and IL-10 (d) in the hippocampus and cortex. Values are mean ± SEM (*n* = 7). ^#^*p* < 0.05, ^###^*p* < 0.001 vs. the control group, ^∗^*p* < 0.05, ^∗∗∗^*p* < 0.001 vs. the model group. (e) The Western blot detection of COX2 and iNOS expression in the hippocampus, and the quantitative analysis of COX2 (f) and iNOS (g) in the hippocampus. (h) The Western blot detection of COX2 and iNOS expression in the cortex, and the quantitative analysis of COX2 (i) and iNOS (j) in the cortex. Values are mean ± SEM (*n* = 6). ^##^*p* < 0.01, ^###^*p* < 0.001 vs. the control group, ^∗^*p* < 0.05, ^∗∗^*p* < 0.01, ^∗∗∗^*p* < 0.001 vs. the model group.

In the assessment of the anti-inflammatory effect of DL0410, the expression levels of TNF-*α*, IL-1*β*, IL-6, and IL-10 were determined by ELISA. In the D-gal-induced group, the levels of TNF-*α*, IL-1*β*, and IL-6 in the hippocampus and cortex were notably upregulated (all *p* value < 0.01). However, DL0410 significantly reduced TNF-*α*, IL-1*β*, and IL-6 levels in the hippocampus and cortex at doses of 3 and 10 mg/kg, respectively (Figures 7(a)–7(c)). IL-10 is an important anti-inflammatory cytokine. In the model group, the expression of IL-10 was significantly increased, and DL0410 could remarkably improve the expression level of IL-10 to a certain extent, indicating a balanced inflammatory system in the DL0410-treated group (Figure 7(d)). Additionally, the expression levels of COX2 and iNOS were detected and analyzed by Western blot. The results showed that systemic chronic treatment with D-gal significantly increased the expression of COX2 and iNOS (both *p* < 0.01). DL0410 significantly reduced the expression of COX2 and iNOS at the dose of 10 mg/kg (Figures 7(e)–7(j)). These data demonstrated that DL0410 decreased the inflammatory cytokines and mediators in the hippocampus and cortex of D-gal-stimulated rats.

### 3.7. DL0410 Inhibited the TLR4/MyD88/NF-*κ*B Signaling Pathway Activation in the Hippocampus and Cortex of D-gal-Stimulated Rats

**Figure 8:**
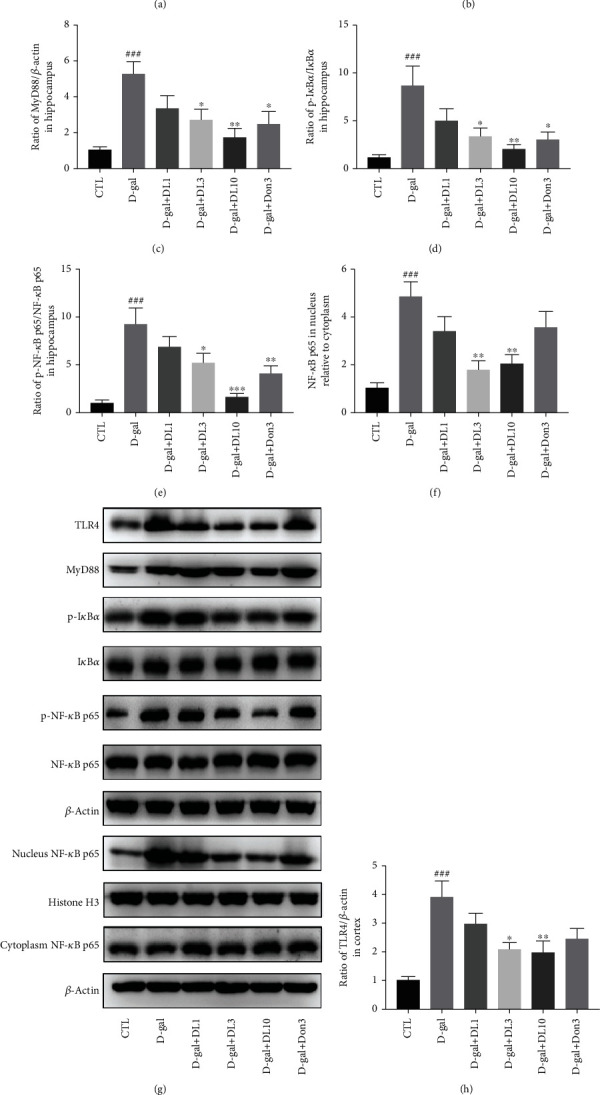
DL0410 inhibited the activation of the TLR4/MyD88/NF-*κ*B signaling pathway in D-gal-stimulated rats. (a) Representative protein band images of TLR4, MyD88, p-I*κ*B*α*, I*κ*B*α*, p-NF-*κ*B p65, NF-*κ*B p65, nucleus NF-*κ*B p65, and cytoplasm NF-*κ*B p65 in the hippocampus. (b) Quantitative analysis of relative expression for TLR4 to *β*-actin in the hippocampus. (c) Quantitative analysis of relative expression for MyD88 to *β*-actin in the hippocampus. (d) Quantitative analysis of relative expression for p-I*κ*B to I*κ*B in the hippocampus. (e) Quantitative analysis of expression ratio for p-NF-*κ*B p65 to NF-*κ*B p65 in the hippocampus. (f) Quantitative analysis of NF-*κ*B p65 (cytoplasm and nucleus) in the hippocampus. (g) Representative images of TLR4, MyD88, p-I*κ*B*α*, I*κ*B*α*, p-NF-*κ*B p65, NF-*κ*B p65, nucleus NF-*κ*B p65, and cytoplasm NF-*κ*B p65 in the cortex. (h) Quantitative analysis of relative expression for TLR4 to *β*-actin in the cortex. (i) Quantitative analysis of relative expression for MyD88 to *β*-actin in the cortex. (j) Quantitative analysis of relative expression for p-I*κ*B to I*κ*B in the cortex. (k) Quantitative analysis of expression ratio for p-NF-*κ*B p65 to NF-*κ*B p65 in the cortex. (l) Quantitative analysis of NF-*κ*B p65 (cytoplasm and nucleus) in the cortex. Values are expressed mean ± SEM of six independent experiments. ^#^*p* < 0.05, ^##^*p* < 0.01, ^###^*p* < 0.001 vs. the control group, ^∗^*p* < 0.05, ^∗∗^*p* < 0.01, ^∗∗∗^*p* < 0.001 vs. the model group.

Pattern recognition receptors, which include Toll-like receptors, are closely related to innate immune activation and can participate in the regulation of the immune response in central nervous system injury. The activation of the TLR4/MyD88 signaling pathway has been widely observed in central nervous system diseases, including AD. As an important transcription factor of inflammatory pathway, NF-*κ*B plays a key role in the development and progression of AD [33]. Studies have reported that stimulation with D-gal caused the TLR4/MyD88/NF-*κ*B signaling pathway activation, thereby activating the inflammatory response and causing continuous damage to neurons. Next, the expression of TLR4, MyD88, I*κ*B*α*, phosphorylated I*κ*B*α*, NF-*κ*B p65, and phosphorylated NF-*κ*B p65 were determined by Western blot. The results showed that the expression of TLR4 (*p* < 0.001 in the hippocampus and cortex) and MyD88 (*p* < 0.001 in the hippocampus and *p* < 0.01 in the cortex) and ratio of p-I*κ*B*α*/I*κ*B*α* (*p* < 0.001 in the hippocampus and cortex) and p-p65/p65 (*p* < 0.001 in the hippocampus and *p* < 0.05 in the cortex) in the D-gal-stimulated group were significantly higher than that of the control group, indicating that the inflammatory process stimulated by D-galactose involved the activation of the TLR4/MyD88/NF-*κ*B signaling pathway. What is more, our immunoblot results showed that D-gal stimulated the nuclear translocation of NF-*κ*B p65 in both the hippocampus and cortex. However, Figure 8 shows that DL0410 treatment significantly inhibited the expression of TLR4 and MyD88, suppressed the phosphorylation of I*κ*B*α* and NF-*κ*B p65, and blocked the nuclear translocation of NF-*κ*B p65, which indicated that the protective effects of DL0410 against D-galactose-induced neuroinflammation might be related to its role in inhibiting the activation of the TLR4/MyD88/NF-*κ*B signaling pathway.

### 3.8. DL0410 Attenuated the Proinflammatory Cytokines Production in LPS-Induced BV2 Cells

**Figure 9:**
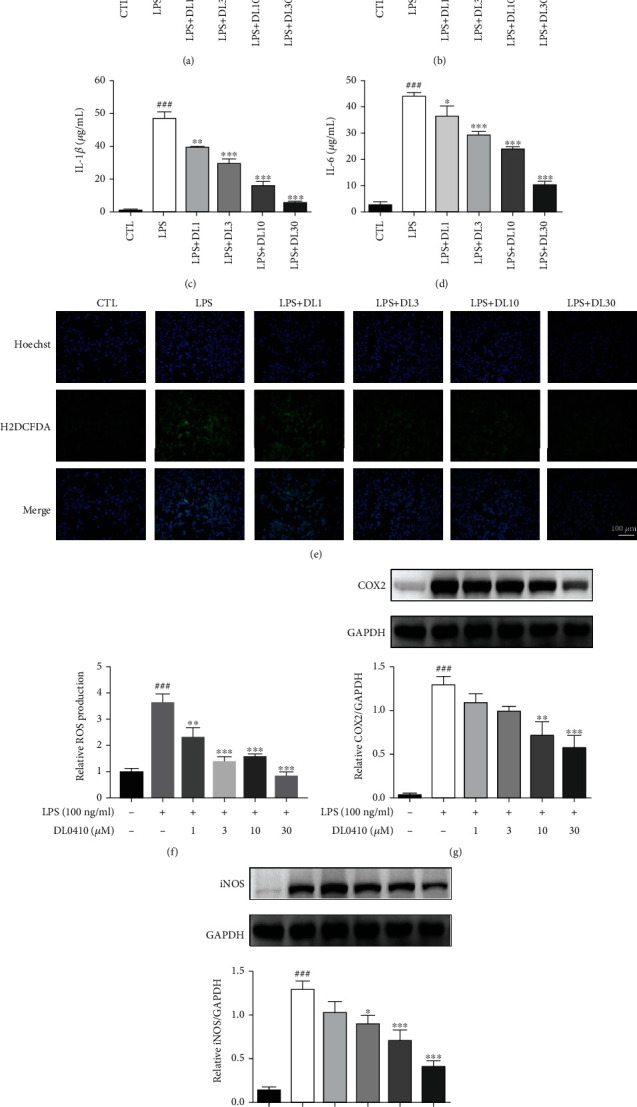
DL0410 decreased proinflammatory cytokine production in LPS-stimulated BV2 cells. The expression of NO (a), TNF-*α* (b), IL-1*β* (c), and IL-6 (d) was detected by ELISA. Intracellular ROS of BV2 microglia was detected by fluorescence intensity after incubation with H2DCFDA (e, f). The protein expression of COX2 (g) and iNOS (h) was measured and analyzed by Western blot. Bar = 100 *μ*m. Values are mean ± SEM of four independent experiments. ^###^*p* < 0.001 vs. the control group, ^∗^*p* < 0.05, ^∗∗^*p* < 0.01, ^∗∗∗^*p* < 0.001 vs. the model group.

Activated microglia stimulated by oxidative damage play a central role in the release of hazardous proinflammatory cytokines and chemokines, leading to neuronal damage and cognitive dysfunction [34]. In order to determine whether DL0410 could decrease the expression levels of proinflammatory cytokines in LPS-stimulated BV2 microglia, we measured the levels of NO, TNF-*α*, IL-1*β*, and IL-6. As shown in Figure 9, the levels of NO, TNF-*α*, IL-1*β*, and IL-6 (all *p* < 0.001) were markedly increased after LPS stimulation. However, DL0410 treatment (1, 3, 10, and 30 *μ*M) significantly reduced the levels of proinflammatory chemokines and cytokines. Also, fluorescent probe H2DCFDA was employed to detect whether DL0410 could exert effects on intracellular ROS production in LPS-stimulated BV2 cells. As revealed by the experimental results, pretreatment with DL0410 successfully decreased the levels of ROS induced by LPS. Statistical analysis of protein bands showed that the expression of COX2 and iNOS was consistent with the results determined in D-gal-induced rats. Collectively, our data demonstrated that DL0410 was capable of inhibiting the secretion and expression of proinflammatory cytokines and chemokines released by activated microglia.

### 3.9. DL0410 Suppressed the TLR4/MyD88/TRAF6/NF-*κ*B Signaling Pathway in LPS-Stimulated BV2 Microglia

**Figure 10:**
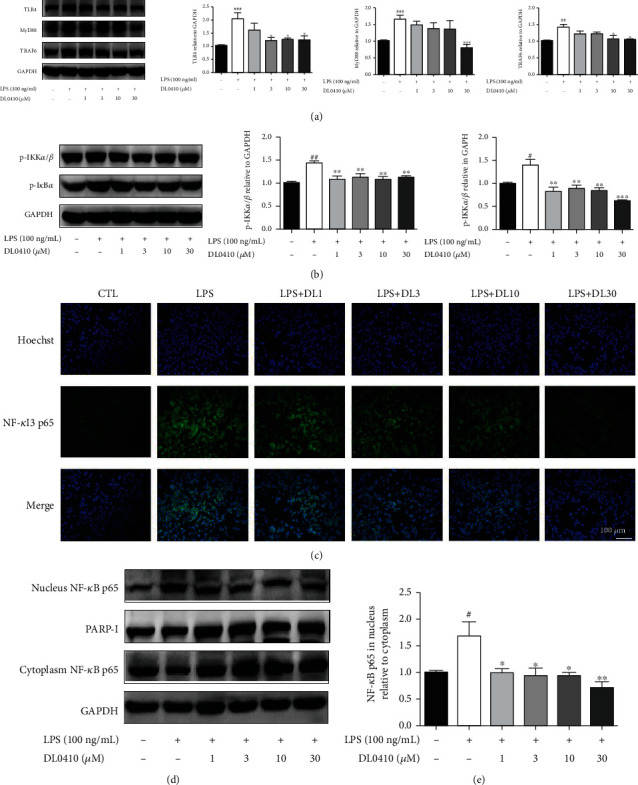
DL0410 suppresses the TLR4/MyD88/TRAF6/NF-*κ*B signaling pathway activation in LPS-stimulated BV2 microglia. Immunoblotting and densitometry analysis of TLR4, MyD88, TRAF6 (a), phospho-I*κ*B*α*, and phospho-IKK*α*/*β* in BV2 cells (b). (c) Immunofluorescence staining for NF-*κ*B p65 (green) and Hoechst (blue) in BV2 microglia. Scale bar = 100 *μ*m. (d, e) Immunoblotting analysis of NF-*κ*B p65 (cytoplasm and nucleus) in LPS-induced BV2 microglia. *n* = 4. All values displayed are mean ± SEM. ^#^*p* < 0.05 or ^##^*p* < 0.01 vs. control, and ^∗^*p* < 0.05 or ^∗∗^*p* < 0.01 vs. LPS-treated cells.

Toll-like receptor 4 (TLR4) is an important member of Toll-like receptors expressed on the surface of mononuclear macrophages that recognizes exogenous substances, taking LPS for example. The interaction of TLR4 with adaptor molecules MyD88 (encoded by myeloid differentiation primary response gene 88) is critical for the activation of downstream signaling pathways and the induction of inflammatory response. Expression of MyD88 is promoted by TLR4 which in turn gives rise to the expression of TNF-receptor associated factor 6 (TRAF6). The NF-*κ*B signaling pathway is one of the downstream pathways of the TLR4/MyD88 pathway. Western blot was carried out to determine whether DL0410 could alter the activation of the TLR4/MyD88/TRAF6 pathway. In BV2 exposed to LPS, the protein levels of TLR4, MyD88, and TRAF6 were significantly upregulated but were downregulated by DL0410 cotreatment, as shown in Figure 10(a).

To gain insight into the involvement of the NF-*κ*B signaling pathway in DL0410-mediated protection effects of LPS-induced neuroinflammatory responses. BV2 cells were pretreated with DL0410 at the given concentration for 2 h, followed by stimulation with LPS (100 ng/mL) for 24 h. As revealed by results in Figure 10, after treatment with LPS, the expression levels of phospho-IKK*α*/*β* and phospho-I*κ*B*α* were significantly increased in activated BV2 microglia. However, DL0410 exposure decreased the levels of phospho-IKK*α*/*β* and phospho-I*κ*B*α* (Figure 10(b)). What is more, LPS stimulation increased the expression of NF-*κ*B p65 subunit in the nucleus while decreased its expression in the cytoplasm. However, the altered ratio of NF-*κ*B p65 in nucleus and cytoplasm was reversed by pretreatment with DL0410. To further confirm the above results, immunofluorescence staining was utilized, and the results also showed that the nucleocytoplasmic translocation of NF-*κ*B p65 induced by LPS was effectively suppressed by DL0410 pretreatment (Figures 10(c)–10(e)).

### 3.10. Putative Binding Mode between the TLR4/MD2 Complex and DL0410

**Figure 11:**
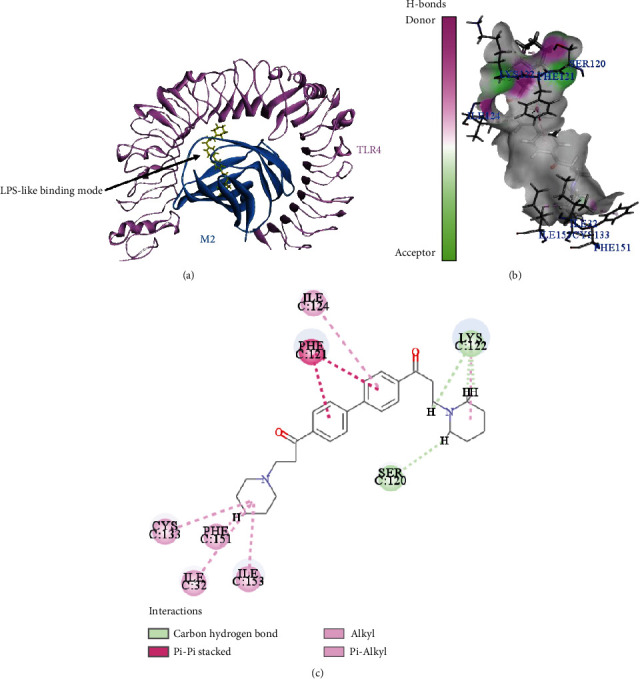
Molecular docking analysis of DL0410 and TLR4/MD2 complex. (a) TLR4 (colored in pink) and MD2 (colored in blue) are represented showing their secondary structure. DL0410 is colored in yellow. (b, c) The receptor–ligand interactions of DL0410 with TLR4/MD2 complex.

A CDOCKER docking study was performed to characterize the binding affinity and putative binding mode to TLR4/MD2 complex of DL0410. Among the 10 receptor-ligand complexes obtained from docking calculation, the one with the lowest binding energy was considered for further analysis. The -CDOCKER energy and -CDOCKER interaction energy between the energetically most stable TLR4/MD2-DL0410 complex were 23.34 and 61.67, respectively, indicating good binding affinity between DL0410 and TLR4/MD2 complex. As shown in Figure 11, DL0410 could be accommodated into the binding pocket of MD2, indicating DL0410 occupying LPS recognition site in the MD2 structure. DL0410 formed two carbon hydrogen bonds with amino acids Ser120 and Lys122, and its biphenyl structure formed Pi–Pi stacked interaction with Phe121. Other interactions of DL0410 binding to TLR4/MD2 complex included Pi–alkyl interaction with Ile124, and interactions with Ile32, Cys133, Phe151, and Ile153.

### 3.11. DL0410 Blocked BBB Dysfunction Injured by D-gal in the Hippocampus and Cortex of Rats

**Figure 12:**
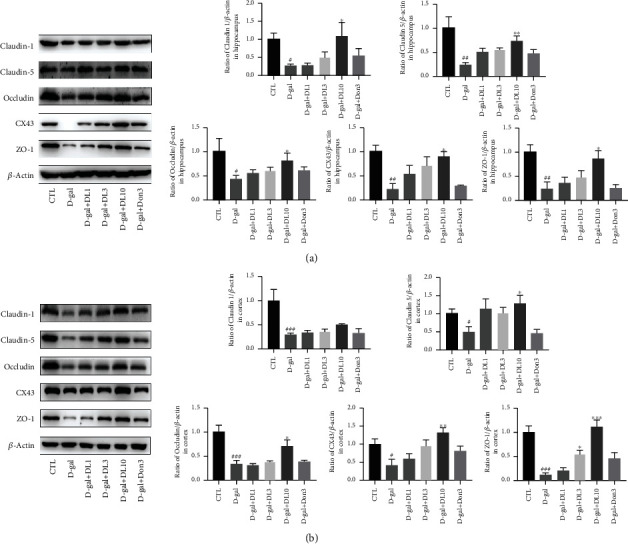
DL0410 increased the expression of tight junction proteins and maintained integrity of blood-brain barrier (BBB) in the hippocampus cortex of rats stimulated by D-gal. (a) The representative images and statistical analysis of relative expression for claudin-1, claudin-5, occludin CX43, and ZO-1 in the hippocampus. (b) The representative images and statistical analysis of relative expression for claudin-1, claudin-5, occludin CX43, and ZO-1 in the cortex. Values are mean ± SEM of six independent experiments. ^#^*p* < 0.05, ^##^*p* < 0.01, ^###^*p* < 0.001 vs. the control group, ^∗^*p* < 0.05, ^∗∗^*p* < 0.01 vs. the model group.

Tight junction proteins including claudins, occludin, and junctional adhesion molecules play an important part in maintaining BBB integrity [35]. Claudins form the paracellular barrier of TJ chain. Occludin is a transmembrane protein in TJ, which can anchor zona occludin (ZO) proteins 1 and 2 in the cytoplasm and plasma membrane of adjacent cells. In addition, occludin is incorporated into claudin-based chain and regulates its permeability. In the research, we studied the effects of DL0410 on the chronic D-gal injection-induced BBB integrity destruction by Western blot. As we can see from Figure 12, the levels of claudin-1, claudin-5, and occludin were all decreased notably compared with the control group, indicating the destruction of BBB integrity in the model group. However, treatment with DL0410 at the dose of 10 mg/kg significantly improved the expression of claudin-1, claudin-5, occludin, CX43, and ZO-1. The increase of protein level suggested that DL0410 could reduce BBB damage injured by D-gal in the hippocampus and cortex.

## 4. Discussion

The present study provides evidence for the protective effects and possible mechanism of DL0410 on the hippocampus and cortex injured by D-gal in rats. The amelioration effects of DL0410 on learning, memory and cognitive function in D-gal-induced rats are associated with the inhibition of oxidative stress and neuroinflammation and the improvement of neuron morphology and synaptic plasticity. The anti-inflammation effect of DL0410 was closely related to the inhibition of the TLR4/MyD88/TRAF6/NF-*κ*B signaling pathway, which was further confirmed in LPS-induced BV2 cells and molecular docking calculation. What is more, this is the first time to find that DL0410 is able to exert positive effects on the integrity of blood-brain barrier.

Long-term treatment with high-dose D-galactose can induce memory and cognitive impairment in rodents. Superoxide anions produced accompanied by D-galactose metabolism directly damage tissues and organs and promote the production of ROS. In addition, D-galactose can also increase the levels of advanced glycosylation end products (AGEs) and MDA through nonenzymatic glycosylation reaction [36], causing oxidative damage and neuroinflammation to brain and leading to neurodegenerative symptoms eventually. Therefore, the D-galactose-induced aging model can partially mimic the human aging process and is widely used for the pharmacodynamic evaluation of antidementia drugs. In the present study, aging rat model stimulated by D-galactose was successfully established and applied to explore the anti-AD effects and possible mechanism of DL0410. A series of tests including the autonomous activity test, Morris water maze test, step-down test, and novel object recognition memory test were adopted to comprehensively evaluate the improvement effects of learning, memory, and cognitive functions of rats. The results showed obvious damage to memory and cognition in D-galactose-administered rats, and DL0410 administration could efficiently improve the rats' performance in behavior test. Furthermore, the therapeutic effect of DL0410 at 10 mg/kg/day could match that of donepezil and was even better than that.

The hippocampus and cortex are the two most important brain regions that affect learning, memory and cognitive function [37, 38]. Neuronal nuclear antigen (NeuN) is a neuronal specific nuclear protein located in the nucleus and cell body of most neurons in vertebrates [39]. It has been reported that the immunoreactivity of NeuN tended to decrease sharply after central nervous system (CNS) injury, and it has been seen as a marker of neurogenesis [40]. Consistent with previous experimental results, we also demonstrated a significant decrease of NeuN staining after D-galactose stimulation compared with the control group, while DL0410 showed a powerful trend to improve this phenomenon, indicating that DL0410 treatment improved injured neuron caused by D-gal in the hippocampus and cortex of rats.

Various pathological mechanisms are thought to be involved in the progression of AD, which would cause neuronal degenerative lesions and death as the final results [41]. The decrease in synaptic plasticity and loss of synapses will directly cause cognitive function dysfunction [42]. Our electron microscopy results demonstrated that in comparison with saline treatment, D-gal treatment significantly decreased the number of synapses in the hippocampus of the rat brain. However, in comparison with D-gal alone, D-gal + DL0410 cotreatment effectively increased the number of synapses. Synaptic plasticity refers to the characteristic that the connection strength between neurons can be adjusted, so as to realize the dynamic change of the signal transmission efficiency, including structural and functional synaptic plasticity [43]. Structural synaptic plasticity is mainly regulated by the change or modification of presynaptic- and postsynaptic-associated protein and is the important major basis for functional synaptic plasticity [44]. PSD95 is the most important and abundant protein on the postsynaptic membrane and is necessary for the activity and stability of the postsynaptic membrane receptor. More and more studies have revealed that synaptic proteins provide a structural integrity to protect synaptic transmission and have extremely close connection with learning and memory ability. Excessive loss of synaptic proteins may result in cognitive dysfunction [45]. In the hippocampus and cortex of D-gal-stimulated rats, we observed significantly decreased expression of PSD95. Promotion of PSD95 expression by DL0410 had a contribution to improving learning and memory ability. These results demonstrated that DL0410 exerts neuroprotection effects by improving synaptic density and synaptic plasticity in the hippocampus and cortex of D-gal-stimulated rats.

D-gal-induced oxidative damage was demonstrated in our study as the higher production and accumulation of AGEs and MDA and destruction to antioxidative system were detected in the model group. Both AGEs and ROS can activate inflammation-related signaling pathways and cause significant neuroinflammation. Neuronal apoptosis and synaptic loss are the common results of oxidative stress and neuroinflammatory damage [46], and also the direct cause of learning and cognitive impairment caused by D-galactose. During the process of brain aging, the increased inflammatory factors are mainly derived from activated astrocytes and microglia. As demonstrated in the staining results of Iba-1 and GFAP, the specific markers of microglia and astrocyte treatment, respectively, with D-gal made microglia and astrocyte overactivated, while DL0410 cut down on the number of Iba-1 and GFAP-positive cells, followed by the reduction of inflammatory factors, including TNF-*α*, IL-1*β*, and IL-6 and increase of anti-inflammatory factor IL-10. Consistent with the results of Dal-induced rats, DL0410 decreased the expression levels of proinflammatory chemokines and cytokines in LPS-stimulated BV2 microglia.

TLR4 is expressed on the neurons, microglia, astrocytes, oligodendrocytes, and vascular endothelial cells in the central nervous system and can be activated by various pathogens and cytokines to regulate the activation of related pathways, expression of enzymes, and inflammatory factors in the process of inflammation. The Myd88-dependent pathway is the most important pathway caused by TLR4 activation. NF-*κ*B is a family of transcription factors that plays an important part in the regulation of inflammatory response [47, 48]. NF-*κ*B p50 and NF-*κ*B p65 exist in the cytoplasm in combination with their inhibitor protein I*κ*B*α* under normal conditions. After IKK*α*/*β* being phosphorylated, I*κ*B*α* is phosphorylated, ubiquitinated, and degraded. Afterwards, the released NF-*κ*B dimers translocate to the nucleus and upregulate genes encoding various proinflammatory proteins or enzymes [49]. NF-*κ*B plays an important role in mediating immune and inflammatory responses. Our present study described a clear decrease by DL0410 on the D-gal-stimulated increased expression of TLR4, MyD88, and phosphorylation of NF-*κ*B p65 and I*κ*B*α* and nuclear translocation of NF-*κ*B, indicating that DL0410 is effective to inhibit the TLR4/MyD88/NF-*κ*B signaling pathway activation. In support of this, we detected the change of the NF-*κ*B pathway in LPS-induced overactivated BV2 microglia. DL0410 suppressed the activation of the TLR4/MyD88/TRAF6/NF-*κ*B signaling pathway and inhibited the nuclear translocation of the NF-*κ*B p65 subunit, providing evidence to support the inhibition of the TLR4/MyD88/NF-*κ*B signaling pathway in the anti-inflammation effects of DL0410.

**Figure 13:**
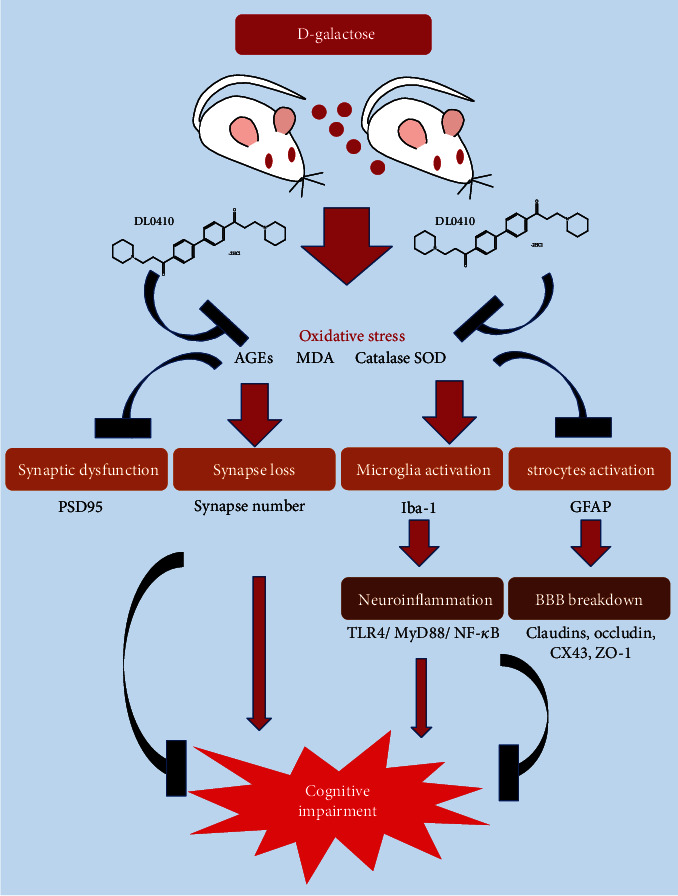
The diagram shows that DL0410 could improve oxidative stressed-induced synaptic dysfunction and synapse loss, suppress microglial and astrocytic activation, inhibit neuroinflammation, and attenuate BBB breakdown to rescue cognitive impairment.

The blood-brain barrier (BBB) is a dynamic structure composed of high-resistance tight junctions (TJ) within the capillary endothelium and makes great contributions to maintaining the homeostasis of the brain and normal function of neurons [50]. Claudins and occludin are the most important constituents of tight junction complexes, responsible for regulating the permeability of epithelia. Astrocytes are of vital importance for maintaining BBB integrity and function, and astrocyte dysfunction could be associated with the early behavioral and cognitive impairments. Under the condition of neuroinflammation, proinflammatory factors, including nitric oxide, reactive oxygen species, and others released from overactivated microglia, can cause damage to the BBB and finally lead to neuron damage. In turn, BBB dysfunction induces the failure of amyloid-*β* transportation and will further trigger neuroinflammation and oxidative stress, forming a vicious circle and bringing about a more serious consequence [51]. BBB breakdown has been demonstrated in the old and late-onset Alzheimer's disease. Our findings offer novel and mechanistic insights into the destruction of D-gal to BBB and the protection of DL0410 for BBB breakdown, as shown in Figure 13.

## 5. Conclusion

Our observations strongly indicated that DL0410 inhibits oxidative damage, neuroinflammation, synaptic plasticity impairment, and BBB integrity damage, resulting in learning, memory, and cognitive improvement in D-gal-induced aging rats. And we provide the first evidence to uncover the inhibition effects of the TLR4/MyD88/NF-*κ*B pathway under inflammatory conditions. Our results offered an additional pharmacological mechanism of DL0410 against D-gal-induced damage and provide evidence for DL0410 as an innovative therapeutic candidate for neuroprotection and antineuroinflammation treatment in AD.

## Data Availability

The data used to support the findings of this study are available from the corresponding author upon request.

## Abbreviations

AD: Alzheimer's disease
PSD95: Postsynaptic density protein 95
TNF-*α*: Tumor necrosis factor *α*
IL-1*β*: Interleukin 1*β*
IL-6: Interleukin 6
IL-10: Interleukin 10
BBB: Blood-brain barrier
NFTs: Neurofibrillary tangles
ROS: Reactive oxygen species
COX2: Cyclooxygenase2
iNOS: Inducible nitric oxide synthase
TJs: Tight junctions
DMSO: Dimethyl sulfoxide
MTT: 3-(4,5-Dimethyl-2-thiazolyl)-2,5-diphenyl-2-H-tetrazolium bromide
BSA: Bovine serum albumin
LPS: Lipopolysaccharide
H2DCFDA: 2′,7′-Dichlorofluorescin diacetate
PFA: Paraformaldehyde
ELISA: Enzyme-linked immunosorbent assay
AGEs: Advanced glycation end products
RIPA: Radio immune precipitation assay
NF-*κ*B: Nuclear factor-kappa B
Iba-1: Calcium-binding adapter molecule 1
NeuN: Neuronal nuclear antigen
TLR4: Toll-like receptor 4
PSD95: Postsynaptic density protein 95
GFAP: Glial fibrillary acidic protein
FBS: Fetal bovine serum
DMEM: Dulbecco's modified Eagle's medium
PBS: Phosphate-buffered saline
NO: Nitric oxide
SD: Sprague-Dawley
D-gal: D-Galactose
DL: DL0410
Don: Donepezil
CTL: Control
NOR: Novel object recognition
SOD: Superoxide dismutase
H_2_O_2_: Hydrogen peroxide
DAPI: 4,6-Diamidino-2-phenylindole
TEM: Transmission electron microscope
PVDF: Polyvinylidene difluoride
MWM: Morris water maze.

## Supplementary Materials

Supplementary Materials Preparation and quantification of DL0410 are shown in supplementary materials.

## References

[1] Atri A. (2019). The Alzheimer’s disease clinical spectrum: diagnosis and management. *The Medical Clinics of North America*.

[2] Veitch D. P., Weiner M. W., Aisen P. S. (2019). Understanding disease progression and improving Alzheimer’s disease clinical trials: recent highlights from the Alzheimer’s disease neuroimaging initiative. *Alzheimers Dement*.

[3] Cummings J., Lee G., Ritter A., Sabbagh M., Zhong K. (2019). Alzheimer’s disease drug development pipeline: 2019. *Alzheimers Dement (N Y)*.

[4] Doig A. J., del Castillo-Frias M. P., Berthoumieu O. (2017). Why is research on amyloid-*β* failing to give new drugs for Alzheimer’s disease?. *ACS Chemical Neuroscience*.

[5] Lin M. T., Beal M. F. (2006). Mitochondrial dysfunction and oxidative stress in neurodegenerative diseases. *Nature*.

[6] Yaribeygi H., Panahi Y., Javadi B., Sahebkar A. (2018). The underlying role of oxidative stress in neurodegeneration: a mechanistic review. *CNS & Neurological Disorders Drug Targets*.

[7] Gao J., Zhou R., You X. (2016). Salidroside suppresses inflammation in a D-galactose-induced rat model of Alzheimer’s disease via SIRT1/NF-*κ*B pathway. *Metabolic Brain Disease*.

[8] Rehman S. U., Shah S. A., Ali T., Chung J. I., Kim M. O. (2017). Anthocyanins reversed D-galactose-induced oxidative stress and neuroinflammation mediated cognitive impairment in adult rats. *Molecular Neurobiology*.

[9] Zhao B., Ren B., Guo R. (2017). Supplementation of lycopene attenuates oxidative stress induced neuroinflammation and cognitive impairment via Nrf2/NF-*κ*B transcriptional pathway. *Food and Chemical Toxicology*.

[10] Kantar Gok D., Ozturk N., Er H. (2015). Effects of rosmarinic acid on cognitive and biochemical alterations in ovariectomized rats treated with D-galactose. *Folia Histochemica et Cytobiologica*.

[11] Tsai S. J., Chiu C. P., Yang H. T., Yin M. C. (2011). s-Allyl cysteine, s-ethyl cysteine, and s-propyl cysteine alleviate beta-amyloid, glycative, and oxidative injury in brain of mice treated by D-galactose. *Journal of agricultural and food chemistry*.

[12] Zhong J., Wang F., Wang Z. (2019). Aloin attenuates cognitive impairment and inflammation induced by d-galactose via down-regulating ERK, p38 and NF-*κ*B signaling pathway. *International Immunopharmacology*.

[13] Sweeney M. D., Zhao Z., Montagne A., Nelson A. R., Zlokovic B. V. (2019). Blood-brain barrier: from physiology to disease and back. *Physiological Reviews*.

[14] Sweeney M. D., Sagare A. P., Zlokovic B. V. (2018). Blood-brain barrier breakdown in Alzheimer disease and other neurodegenerative disorders. *Nature Reviews. Neurology*.

[15] Yamazaki Y., Kanekiyo T. (2017). Blood-brain barrier dysfunction and the pathogenesis of Alzheimer’s disease. *International Journal of Molecular Sciences*.

[16] Qin W., Li J., Zhu R. (2019). Melatonin protects blood-brain barrier integrity and permeability by inhibiting matrix metalloproteinase-9 via the NOTCH3/NF-*κ*B pathway. *Aging (Albany NY)*.

[17] Cai Z., Qiao P. F., Wan C. Q., Cai M., Zhou N. K., Li Q. (2018). Role of blood-brain barrier in Alzheimer’s disease. *Journal of Alzheimer's Disease*.

[18] Fang J., Li Y., Liu R. (2015). Discovery of multitarget-directed ligands against Alzheimer’s disease through systematic prediction of chemical-protein interactions. *Journal of Chemical Information and Modeling*.

[19] Zhou D., Zhou W., Song J. K. (2016). DL0410, a novel dual cholinesterase inhibitor, protects mouse brains against A*β*-induced neuronal damage via the Akt/JNK signaling pathway. *Acta Pharmacologica Sinica*.

[20] Lian W., Fang J., Xu L. (2017). DL0410 ameliorates memory and cognitive impairments induced by scopolamine via increasing cholinergic neurotransmission in mice. *Molecules*.

[21] Yang R. Y., Zhao G., Wang D. M. (2015). DL0410 can reverse cognitive impairment, synaptic loss and reduce plaque load in APP/PS1 transgenic mice. *Pharmacology, Biochemistry, and Behavior*.

[22] Zhang B., Zhao J., Wang Z., Xu L., Liu A., du G. (2020). DL0410 attenuates oxidative stress and neuroinflammation via BDNF/TrkB/ERK/CREB and Nrf2/HO-1 activation. *International Immunopharmacology*.

[23] Tian H., Ding N., Guo M. (2019). Analysis of learning and memory ability in an Alzheimer’s disease mouse model using the Morris water maze. *Journal of Visualized Experiments*.

[24] Guo H., Cheng Y., Wang C. (2017). FFPM, a PDE4 inhibitor, reverses learning and memory deficits in APP/PS1 transgenic mice via cAMP/PKA/CREB signaling and anti-inflammatory effects. *Neuropharmacology*.

[25] Aydın A. F., Küçükgergin C., Çoban J. (2018). Carnosine prevents testicular oxidative stress and advanced glycation end product formation in D-galactose-induced aged rats. *Andrologia*.

[26] Hambright W. S., Fonseca R. S., Chen L., Na R., Ran Q. (2017). Ablation of ferroptosis regulator glutathione peroxidase 4 in forebrain neurons promotes cognitive impairment and neurodegeneration. *Redox Biology*.

[27] Broadhead M. J., Horrocks M. H., Zhu F. (2016). PSD95 nanoclusters are postsynaptic building blocks in hippocampus circuits. *Scientific Reports*.

[28] Sadigh-Eteghad S., Geranmayeh M. H., Majdi A., Salehpour F., Mahmoudi J., Farhoudi M. (2018). Intranasal cerebrolysin improves cognitive function and structural synaptic plasticity in photothrombotic mouse model of medial prefrontal cortex ischemia. *Neuropeptides*.

[29] Lu J., Wu D. M., Hu B. (2010). Chronic administration of troxerutin protects mouse brain against d-galactose-induced impairment of cholinergic system. *Neurobiology of Learning and Memory*.

[30] Kaur D., Sharma V., Deshmukh R. (2019). Activation of microglia and astrocytes: a roadway to neuroinflammation and Alzheimer’s disease. *Inflammopharmacology*.

[31] Phatnani H., Maniatis T. (2015). Astrocytes in neurodegenerative disease. *Cold Spring Harbor Perspectives in Biology*.

[32] Norden D. M., Trojanowski P. J., Villanueva E., Navarro E., Godbout J. P. (2016). Sequential activation of microglia and astrocyte cytokine expression precedes increased Iba-1 or GFAP immunoreactivity following systemic immune challenge. *Glia*.

[33] Seo E. J., Fischer N., Efferth T. (2018). Phytochemicals as inhibitors of NF-*κ*B for treatment of Alzheimer’s disease. *Pharmacological Research*.

[34] Rangaraju S., Dammer E. B., Raza S. A. (2018). Identification and therapeutic modulation of a pro-inflammatory subset of disease-associated-microglia in Alzheimer’s disease. *Molecular Neurodegeneration*.

[35] Berndt P., Winkler L., Cording J. (2019). Tight junction proteins at the blood-brain barrier: far more than claudin-5. *Cellular and Molecular Life Sciences*.

[36] Zeng L., Lin L., Peng Y. (2020). l-Theanine attenuates liver aging by inhibiting advanced glycation end products in d-galactose-induced rats and reversing an imbalance of oxidative stress and inflammation. *Experimental Gerontology*.

[37] Rothschild G., Eban E., Frank L. M. (2017). A cortical-hippocampal-cortical loop of information processing during memory consolidation. *Nature Neuroscience*.

[38] Yavas E., Gonzalez S., Fanselow M. S. (2019). Interactions between the hippocampus, prefrontal cortex, and amygdala support complex learning and memory. *F1000Research*.

[39] Melo K. P., Silva C. M., Almeida M. F. (2019). Mild exercise differently affects proteostasis and oxidative stress on motor areas during neurodegeneration: a comparative study of three treadmill running protocols. *Neurotoxicity Research*.

[40] Lu P., Ceto S., Wang Y. (2017). Prolonged human neural stem cell maturation supports recovery in injured rodent CNS. *The Journal of Clinical Investigation*.

[41] Mullane K., Williams M. (2018). Alzheimer’s disease (AD) therapeutics-1: repeated clinical failures continue to question the amyloid hypothesis of AD and the current understanding of AD causality. *Biochemical Pharmacology*.

[42] Varbanov H., Dityatev A. (2017). Regulation of extrasynaptic signaling by polysialylated NCAM: impact for synaptic plasticity and cognitive functions. *Molecular and Cellular Neurosciences*.

[43] Magee J. C., Grienberger C. (2020). Synaptic plasticity forms and functions. *Annual Review of Neuroscience*.

[44] Tian X., Liu Y., Ren G. (2016). Resveratrol limits diabetes-associated cognitive decline in rats by preventing oxidative stress and inflammation and modulating hippocampal structural synaptic plasticity. *Brain Research*.

[45] Wu Q., Sun M., Bernard L. P., Zhang H. (2017). Postsynaptic density 95 (PSD-95) serine 561 phosphorylation regulates a conformational switch and bidirectional dendritic spine structural plasticity. *The Journal of Biological Chemistry*.

[46] Kinney J. W., Bemiller S. M., Murtishaw A. S., Leisgang A. M., Salazar A. M., Lamb B. T. (2018). Inflammation as a central mechanism in Alzheimer’s disease. *Alzheimers Dement (N Y)*.

[47] Liu T., Zhang L., Joo D., Sun S. C. (2017). NF-*κ*B signaling in inflammation. *Signal Transduction and Targeted Therapy*.

[48] Wang L., Liu H., Zhang L., Wang G., Zhang M., Yu Y. (2017). Neuroprotection of dexmedetomidine against cerebral ischemia-reperfusion injury in rats: involved in inhibition of NF-*κ*B and inflammation response. *Biomolecules & therapeutics*.

[49] Mitchell J. P., Carmody R. J. (2018). NF-*κ*B and the transcriptional control of inflammation. *International Review of Cell and Molecular Biology*.

[50] Montagne A., Zhao Z., Zlokovic B. V. (2017). Alzheimer’s disease: a matter of blood-brain barrier dysfunction?. *The Journal of Experimental Medicine*.

[51] Janyou A., Wicha P., Jittiwat J., Suksamrarn A., Tocharus C., Tocharus J. (2017). Dihydrocapsaicin attenuates blood brain barrier and cerebral damage in focal cerebral ischemia/reperfusion via oxidative stress and inflammatory. *Scientific Reports*.

