# Supplementary material for: DL0410 Alleviates Memory Impairment in D-Galactose-Induced Aging Rats by Suppressing Neuroinflammation via the TLR4/MyD88/NF-κB Pathway
Source: Oxid Med Cell Longev. 2021 Oct 4;2021:6521146. doi: 10.1155/2021/6521146 (PMC8510815; doi:10.1155/2021/6521146)
Supplement: Supplementary Materials — Preparation and quantification of DL0410 are shown in supplementary materials. [file 6521146.f1.docx]

**Preparation and quantification of DL0410**

Synthetic pathway:


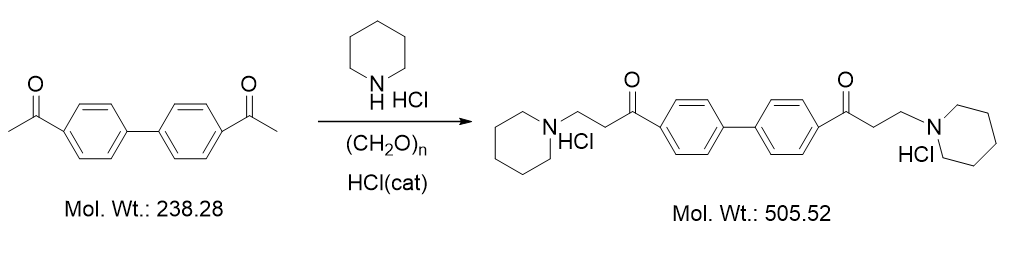


Preparation and quantification of reference substances:

60 g of reference substances were acquired after samples (80.0 g, No.20140901) were recrystallized by isopropanol water solvent mixtures. Results analyzed by HPLC showed the purity of the reference material was 99.84% with normalization of peak areas method.

Instrument used for HPLC: an Agilent 1200 series (USA);

Detection conditions:

Column: Eclipse XDS C18 (4.6×250 mm, 5µm);

Mobile phase: A: an aqueous 0.05% phosphate buffer, B: methanol, gradient elution;

Column temperature: 30 ℃;

Flow rate: 1.0 mL/min;

Injection volume: 30 µL;

Analytical time: 22 min;

Detection wavelength: 300 nm

|  | Retention time | Area | Height | % Area |
| --- | --- | --- | --- | --- |
| 1 | 7.217 | 81563492 | 1894005 | 99.84 |
| 2 | 10.953 | 131083 | 4543 | 0.16 |
